# Editorial: Novel Advances in Allergy Diagnosis and Treatment

**DOI:** 10.3389/fimmu.2021.662699

**Published:** 2021-04-13

**Authors:** Simon Blank, Christiane Hilger

**Affiliations:** ^1^ Center of Allergy and Environment (ZAUM), Technical University of Munich, Faculty of Medicine and Helmholtz Center Munich, German Research Center for Environmental Health, Member of the German Center of Lung Research (DZL), Member of the Immunology and Inflammation Initiative of the Helmholtz Association, Munich, Germany; ^2^ Department of Infection and Immunity, Luxembourg Institute of Health (LIH), Esch-sur-Alzette, Luxembourg

**Keywords:** allergen, allergen-specific immunotherapy (AIT), allergy, allergy diagnosis, atopic diseases

The term “allergy” was coined by Clemens von Pirquet in 1906 to describe a general change of the organisms’ reactivity in quality, quantity and time, including hyper- and hyposensitivity reactions to exogenous substances (allergens) which are also depending on endogenous factors (1). Today, the word “allergy” is associated with an abnormal, adaptive immunologic hypersensitivity reaction to non-infectious environmental substances. The most common manifestations of allergic diseases are IgE-mediated hypersensitivity reactions which in the last decades have become a major health problem as already more than one quarter of the population in industrialized countries is affected and prevalence is further rising (2). Allergen sources include a wide variety of environmental substances such as pollen, house dust mites, animal dander, foods, drugs or insect venoms and the disease can manifest itself e.g. as rhinitis, conjunctivitis, chronic asthma, urticaria or even life-threatening anaphylaxis (2, 3). Long before the availability of anti-allergic drugs, Leonard Noon demonstrated in 1911 that prophylactic inoculation with grass pollen extract was efficient in suppressing symptoms of hay fewer (4). Since that time, allergen-specific immunotherapy (AIT) remains the only available curative treatment for allergic patients. Nevertheless, in recent times, several novel approaches aiming at enhancing therapeutic efficacy and diagnostic accuracy have been developed. Moreover, the ongoing elucidation of immunological mechanisms of allergic sensitization, disease progression and tolerance induction to allergens will facilitate the development of new preventive and therapeutic strategies against allergy (5).

Although AIT is a well-established disease-modulating treatment for IgE-mediated allergic diseases, the induction of immune tolerance is an evolving area that is still not sufficiently understood. Zissler and Schmidt-Weber give a comprehensive overview on immunological changes during AIT and their usefulness as biomarker for monitoring and predicting therapeutic success. They discuss that clinical allergen tolerance depends on multiple mechanisms across different immune cell and tissue compartments. Hence, it is likely that only combinations or ratios of gene expression levels are promising to achieve predictive value and definition of helpful biomarkers. Outstanding effective tolerance induction can be achieved by AIT of Hymenoptera venom-allergic patients. Blank et al. describe how the classification of venom-allergic patients into different disease endotypes and phenotypes applying available biomarkers and diagnostic tolls can provide therapeutic guidance and strengthen personalized treatment strategies and precision medicine. Along the same lines, Czolk et al. provide an overview on the immune basis for phenotype variations in peanut-allergic individuals. They discuss that deep immune phenotyping and multi-omics technologies can build a reliable basis for novel insights into disease pathophysiology and identification of biomarkers or biomarker signatures predictive for reaction phenotypes. This knowledge shall advance the stratification of individuals prior to selection for oral immunotherapy or early food introduction for prevention.

Efficacy and safety of AIT relies on the type of allergy and the disease status of the patient. Several approaches aiming at enhancing therapeutic efficacy and reducing side-effects have been developed or are in the scientific pipeline. For instance, the use of adjuvants may optimize the immunological response to AIT in the most appropriate way for a specific disease manifestation. The current status of the application of the adjuvant Microcrystalline Tyrosine (MCT) and adjuvant systems comprising MCT and Monophosphoryl Lipid A (MPL) in AIT is reviewed by Heath et al. Montamat et al. describe the potential of CpG oligonucleotides as adjuvant for AIT with a focus on dose- and concentration-dependent effects that are crucial for the induction of inflammatory or tolerogenic responses. A broader overview on existing and promising new candidates for formulations of AIT of human and veterinary patients, including adjuvants, immunomodulators, physical packaging, conjugates and combinations thereof to modify allergenic proteins, making them safer, and more efficacious in AIT, is given by Pali-Schöll et al.

There are some disadvantages associated with AIT when using complete protein extracts from allergenic sources. These include among others, problems with standardization and allergen stability, as well as the fact that the patients will be treated with a whole cocktail of allergens and non-allergenic proteins, while having a specific IgE-profile (6). AIT based on recombinant hypoallergenic allergens has raised attention, especially in allergic diseases which are triggered by one dominant allergen as e.g. birch pollen allergy. Aglas et al. demonstrate a robust IgG immune response against a hypoallergenic variant of the major birch pollen allergen Bet v 1 in rats that efficiently blocks human IgE-binding to the wild-type allergen, thereby demonstrating its potential therapeutic value in AIT. Flicker et al. discuss the potential benefit of nanobodies, single domain antibodies with several superior properties compared to conventional antibodies, for passive treatment of IgE-mediated allergy. The study by Krause et al. presents the development of a high throughput analytical platform for unbiased IgE target epitope detection. Such epitopes may represent interesting candidates for diagnosis as well as therapy. Pomés et al. give a comprehensive overview of state-of-the-art approaches to analyze the interaction between IgE-antibodies and corresponding allergen epitopes. This information on antigenic determinants will facilitate the design of hypoallergens for AIT and further elucidate fundamental mechanisms of the IgE immune response.

Evolutionary old responses against helminth parasites closely resemble the patho-mechanisms that drive allergic diseases. This implies that studying the helminth-host-interaction may contribute to novel strategies for fighting against the rise of allergic diseases. Bohnacker et al. review the protective role of helminths in asthma and allergy. The immunomodulatory properties of helminth molecules make them promising candidates to become the next generation of biotherapeutics for the treatment of type 2 inflammatory disorders. Also commensal microbes have a tremendous impact on human health and recent evidence indicates that the susceptibility to food allergy is critically linked to microbial dysbiosis. Kreft et al. give an overview on this important research area and explore future directions for a potential microbial therapy of food allergy. In most cases, proteins are recognized as allergens, although the relevance of carbohydrate-specific antibodies as mediators of IgE-mediated allergy was described decades ago. Hils et al. review the historical development of carbohydrate-allergen-research with a particular focus on clinical and immunological features of the alpha-gal syndrome, the underlying feature of red meat allergy.

Many immune mechanisms of allergic diseases were uncovered by applying animal models of allergy which also often build the basis for the development of anti-allergic treatments. Alessandrini et al. give an overview of currently used type 2 and non-type 2 rodent asthma models and discuss the limits of extrapolation from mice to humans. Currently available biological therapies applying monoclonal antibodies to treat asthma and their possible effects on airway remodeling are reviewed by Kardas et al. There is growing evidence that allergic diseases may develop over lifetime from atopic dermatitis and food allergy in infancy to gradual development into allergic rhinitis and allergic asthma in childhood. Yang et al. give an overview on this so called atopic march and discuss new perspectives for prevention and treatment of atopic diseases that are provided by this concept.

Importantly, the success of AIT depends on an accurate allergy diagnosis that aims at identifying the primary allergen source and risk factors for disease severity. Based on recombinant allergens, molecular or component-resolved allergy diagnosis was introduced into clinical practice and allowed dissecting the molecular sensitization profiles of allergic patients. Huang et al. demonstrate that detection of IgE- and IgG-reactivity to a panel of respiratory allergens micro-arrayed onto silicon elements is more sensitive than glass-based chips. Furthermore, they discuss the advantages of silicon-based allergen microarrays and how this technology will allow addressing hitherto unmet needs in micro-array-based molecular allergy diagnosis. Another powerful tool and sensitive marker that can be used to detect clinically relevant allergy is the basophil activation test (BAT). Eberlein gives an overview on this diagnostic approach and on how the BAT provides information on the severity of an allergic reaction and its use to monitor immunotherapy and desensitization. Barbaud et al. report a multi-center study demonstrating that standardization of intradermal tests with drugs reduces variability and enables a more reliable comparison of results between individuals and centers. Nowadays, it becomes evident that also clinical decision support systems (CDSS) reinforce health care professionals in taking informed decisions during their clinical routine. Dramburg et al. give an overview on existing tools, new developments and novel concepts and discuss the potential of digital CDSS in improving prevention, diagnosis and monitoring of allergic diseases.

In conclusion, state-of-the-art and novel promising diagnostic, therapeutic and basic concepts that will help to fight the global burden of allergic diseases have been introduced and discussed in this Research Topic. Recent advances in diagnostic strategies and biomarker development have greatly improved diagnostic sensitivity and specificity and demonstrate rising potential for detecting clinically relevant allergy and risk factors for severe reactions as well as to predict and monitor therapeutic success in the future. Furthermore, the development of new therapeutic formulations and the uncovering of basic mechanisms of immune tolerance increasingly will contribute to more efficient AIT regimens, even in difficult to treat patients, and presumably also to completely new therapy concepts for allergy.

## Author Contributions

SB and CH wrote the manuscript and contributed equally to the editorial process for this collection. All authors contributed to the article and approved the submitted version.

## Funding

This work was supported by the Helmholtz Association, Future Topic “Immunology and Inflammation” (ZT-0027) to SB.

## Conflict of Interest

SB reports nonfinancial support from ALK-Abelló, grants, personal fees and nonfinancial support from Bencard Allergie GmbH, personal fees from Teomed AG, grants from Leti Pharma, grants and personal fees from Thermo Fisher Scientific, grants from Allergy Therapeutics, outside the submitted work. CH reports grants from Laboratoires Réunis, Luxembourg, and non-financial support from Macro Array Diagnostics, Vienna, outside the submitted work.
